# Supplementary figures and images for: First eight residues of apolipoprotein A-I mediate the C-terminus control of helical bundle unfolding and its lipidation
Source: PLoS One. 2020 Jan 16;15(1):e0221915. doi: 10.1371/journal.pone.0221915 (PMC6964839; doi:10.1371/journal.pone.0221915)

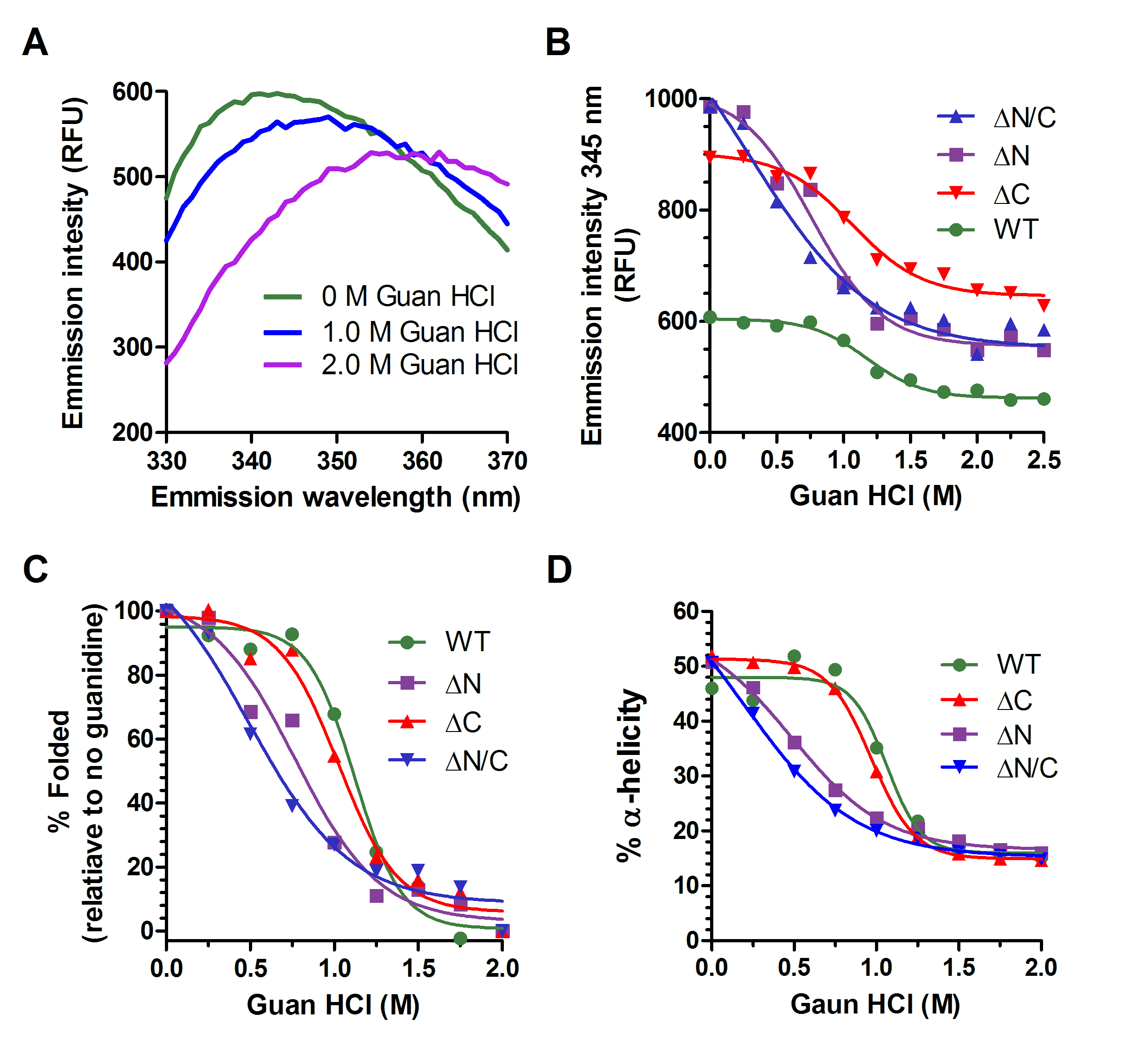

Supplement: S1 Fig — A. An example of WT apoA1 Trp fluorescence emission scans at 0 (green), 1.0 (blue), and 2.0 M (purple) guanidine hydrochloride. B. Guanidine unfolding of the WT (green), ΔN (purple), ΔC (red), and ΔN/C (blue) apoA1 isoforms assessed using the Trp fluorescence emission at 345 nm. The EC50 = 1.12, 0.76, 1.06, and 0.31 M guanidine for unfolding of the WT, ΔN, ΔC, and ΔN/C apoA1 isoforms, respectively. C. The % folded using the single wavelength emission data at 345 nm was calculated by normalizing the data to the 0 M guanidine, and considering full unfolding at 2.0 M guanidine. D. Circular dichroism was used to access % α-helicity at increasing guanidine concentrations. The EC50 = 1.06, 0.48, 0.97, 0.20 M guanidine for the WT, ΔN, ΔC, and ΔN/C isoforms, respectively. (TIF) [file pone.0221915.s001.tif]
