# Supplementary material for: First eight residues of apolipoprotein A-I mediate the C-terminus control of helical bundle unfolding and its lipidation
Source: PLoS One. 2020 Jan 16;15(1):e0221915. doi: 10.1371/journal.pone.0221915 (PMC6964839; doi:10.1371/journal.pone.0221915)
Supplement: Attachment — Submitted filename: Response to Reviewers.docx [file pone.0221915.s003.docx]

We thank the reviewers for their careful reading of our manuscript and for their suggestions to improve manuscript presentation and clarity. Below are our point by point responses to the reviewer comments. Some other minor changes or corrections were made as well, as can be seen in the track changes mode version submitted.

**Reviewer #1:** Brubaker et al. present a characterization of apoA1 lipidation mechanism via unfolding of the N-terminal α-helices. Although the structural information on apoA1 is rather abundant including available structures of different conformations of the protein - particularly from the Davidson group - the authors provide convincing evidence that the very first N-terminal residues including Trp8 have a functional role solubilizing DMPC lipids or in cholesterol transport. Based on fluorescence measurements, the authors propose a mechanism by which unfolding of an N-terminal helix bundle is required for apoA1 lipidation. The experiments are performed with care and the data analyzed correctly. However, major concerns arise from the experiments shown.

1.- Figure 1 shows that deletion of the C-terminus (ΔC mutant) blocks apoA1 functionality, but the additional deletion of the N-terminus (ΔN/ΔC mutant) recovers lipid binding activity. This evidence goes in line with the model of monomeric, full-length apoA1 structure (Melchior et al NSMB 2017), in which the disordered C-terminus caps the N-terminal helix bundle. Lipid binding to the C-terminal domain of apoA1 induces a conformational switch on this region (Oda et al NSMB 2003) that favors the unfolding of the N-terminal helix bundle. The authors present compelling functional data supporting the hypothesis that unfolding of the N-terminal helical bundle is critical for functional lipidation (especially Figure 1B). Then the authors aim to follow the chemical and thermal unfolding of the different variants of apoA1 by monitoring the change in fluorescence maxima (Figure 1C, D). While full-length and ΔC apoA1 constructs contain 4 Trp residues, ΔN variants contain three Trp residues. More importantly, the structure (for instance, PDB code 3r2p) shows that, precisely, helix 1 clusters helices 2-5 in the bundle. Therefore, removal of helix 1 –assuming that the rest of the bundle remains folded- would expose the remaining Trp residues (Trp50, Trp72, Trp108) to the solvent in monomeric ΔN constructs. In this scenario, it is surprising that the authors observe a change in the fluorescence maxima upon chemical and thermal denaturation. One possible explanation would be that removal of helix 1 promotes oligomerization, in such way that the authors are actually following dissociation of oligomers in ΔN and ΔN/ΔC constructs in Figure 1C, D. To discard this possibility, the authors must show that ΔN, ΔC and ΔN/ΔC constructs are monomeric in the experimental conditions used. Full-length apoA1 is already shown to be monomeric in the SDS-PAGE presented in Figure 3B.

**Response:** ApoA1 purified from plasma and renatured from urea or guanidine at >0.1 mg/ml forms a dimer (calculated MW = 56.158 kDa) that runs at ~7.1 nm in non-denaturing gradient gel electrophoresis (NDGGE), approximating the 66 kDa molecular weight of the 7.1 nm protein standard. We added a new figure (Figure 1A), showing NDGGE of his-tagged WT, ΔN, ΔC, and ΔN/C apoA1 isoforms renatured from guanidine at ~0.4 mg/ml. This gel shows that all four of these isoforms run close to the predicted size of dimers. The modified text reads “Non-denaturing gradient gel electrophoresis demonstrated migration as dimers for all four proteins, with calculated diameters of 7.34, 6,82, 6.71, and 6.38 nm for the WT, ΔN, ΔC, and ΔN/C isoforms, respectively, closely agreeing with the theoretical diameters based on the changes in isoform mass (Figure 1A)”. Thus, removal of N-terminal 43 does not promote oligomerization as suspected by reviewer 1. Removing this N-terminus only partially exposes the 3 remaining Trp residues as observed by the slight shift in WMF at 0 M guanidine, as stated in our text: “In addition, in the absence of guanidine, both N-terminal deletion isoforms have higher basal WMF than the WT and ΔC isoforms.” However, the N-terminal deleted forms can still be denatured by guanidine, (Figure 1B) showing that the loss of the N-terminus does not cause complete unfolding of the hairpin, just destabilizing this structure.

2.- Following protein denaturation by the change in fluorescence maxima is not a quantitative method to determine changes in the populations of folded and unfolded conformers. Different degrees of compaction in unfolded proteins will yield fluorescence maxima at different wavelengths. Authors should monitor the change in fluorescence in a single wavelength to follow protein unfolding.

**Response:** The shift in WMF is in fact an ideal method for quantitatively examining the hydrophobic vs. hydrophilic environment of endogenous Trp residues, as it is not dependent upon protein concentration (and thus can easily be used to compare different protein isoforms even if their concentrations are subtly different) and its use as a quantitative method to study apoA1 unfolding has been championed by Michael Phillips and used many other investigators to study apoA1 structure. We adding a new supplemental figure, described in the text: “Examples of the Trp emission spectra for the WT isoform at 0, 1, and 2 M guanidine are shown in Supplemental Figure S1A.”. In addition we added another analysis method, described in the text: “An alternate measure of unfolding using the Trp emission ratio at 355/350 nm yielded similar results as the WMF plot; although, the unfolding EC50 values were shifted to lower guanidine, and the ΔC was harder to unfold than the WT isoform using this measure (Supplemental Figure S1B, EC50 = 0.85 and 1.09 M guanidine for the WT and ΔC isoforms, respectively).”

3.- Proteins were purified in mild denaturing conditions. Proper refolding in the experimental conditions used should be assessed (by Circular Dichroism or comparable techniques). This is particularly important for the ΔN, ΔC and ΔN/ΔC deletion mutants. This point is additionally related to the previous point, since CD would not only be informative on the proper folding of the deletion mutants, but could also be very helpful to characterize protein unfolding upon thermal or chemical denaturation (by monitoring the change in ellipticity in a single wavelength, usually a minima characteristic of α-helical structures).

**Response:** We performed CD on these isoforms and revised the text as follows: “We previously demonstrated by following α-helicity using circular dichroism that all four isoforms can be rapidly unfolded by guanidine, and rapidly refolded upon guanidine dilution (16). We performed circular dichroism on these four isoforms and determined α-helicity of 44.2%, 49.9%, 56.2%, and 50.1% for the WT, ΔN, ΔC, and ΔN/C isoforms, respectively, which are similar to values previously reported for similar recombinant apoA1 isoforms (19, 20).” However, α-helicity measurements by CD are very sensitive to protein concentration, and even within one lab, the levels of α-helix in apoA1 is variable. In addition, CD follows the secondary structure, but the present study is more concerned with tertiary structure; thus, the use of WMF to follow the Trp environment is particularly useful for our goals.

4.- Figure 1A shows that ΔN and ΔN/ΔC mutants partially recover activity. The authors should state that the mutants “only partially” recover the activity (in Figure legend 1A page#9). Do the authors have any explanation for the observed difference between WT and ΔN and ΔN/ΔC mutants in solubilizing DMPC (only about 50% recovery)?

**Response:** In the revised text we now state: “We confirmed prior studies that the WT, ΔN, and ΔN/C isoforms were competent to clear DMPC MLVs, albeit with less activity for the ΔN, and ΔN/C isoforms, while the ΔC isoform was incapable of clearing these liposomes (Figure 1B) (10, 12).” The title to Figure 1 is changed to: “Figure 1. ApoA1 ΔN/C double deletion partially or fully restores activity of the ΔC isoform.”

Minor concerns:

1.- Figure 1A should contain error bars.

**Response:** In the revised figure, this is Figure 1B, and we included the error bars.

2.- Authors state that “the C-terminus stabilized the folded state” based on fluorescence data. However, the ΔC mutant shows much lower change in fluorescence maxima upon thermal denaturation, probably because of the high entropic contribution of the disordered C-terminal region present in WT. Thus, it seems that the C-terminus indeed destabilized the folded state, since in its absence the change in unfolded populations is minor compared to the WT.

**Response:** This was our typographical mistake, we corrected this in the text to read: “***the deletion of*** the C-terminus stabilized the folded state.”

3.- Text should be carefully edited: last line in the Introduction reads “who’s” and it should be “whose”. 3 lines before the last in page#5: Remove “of” in “counting radioactivity to measure of cholesterol”… Line 8, page 10, should read “completely” rescued instead of “completed”

**Response.** Thanks, we corrected these mistakes in the revised text.

4.- Abbreviations should be consistent. The protein is called apoA1 and even apoAI. The C-terminal deletion mutant is termed ΔC and also in page#11 it is called Δ183-243 apoA1.

**Response:** We corrected these abbreviations to consistently use apoA1 and ΔC.

5.- Labeling of the helices in Figures 6C-D would be appreciated.

**Response:** We added the helix numbering to these panels. There are more than one way to number the helices, we decided to match the nomenclature used by the “consensus” model and modified the legend accordingly.

**Reviewer #2:** This study aims to characterize determinants of helical bundle unfolding necessary for lipidation of apolipoprotein A-1 that is mediated by the first eight residues that interact with C-terminus of the protein. The approach uses a Cholesterol Efflux and liposome clearance assay that are now standard to estimate the functionally of the apoA1. In addition to both assays, the authors perform denaturation assay in presence of Guanidium Chloride and Temperature influence. The results suggest that the first eight residues, and specially Trp8, are essential to control the unfolding of the helical bundle necessary for further ApoA1 lipidation. Several published work, spanning the last 20 years, have presented evidence consistent with the current manuscript, but nonetheless, this paper adds a little bit more details and tend to clarify some tenets of apoA1 lipidation and its mechanism.

Taken together the results of the paper are suitable for publication in Plos One. However, there are some points that should be amended or clarified before the manuscript may be accepted. These are detailed in the following:

Major revisions:

- Authors used WMF for unfolding experiments as a red shift is produced when the ApoA1 Trp present in the structure go from a hydrophobic to an aqueous environment during the unfolding process. When Trp are exposed to the hydrophilic environment, an increase of the intensity of the fluorescence is generally observed. The manuscript would gain reproducibility if the authors could provide at least one Trp fluorescence spectrum where the red shift and intensity changes are observed, at least for the Wt and ΔC and ΔN isoforms

**Response:** As described above, we added a new supplemental figure, described in the text: “Examples of the Trp emission spectra for the WT isoform at 0, 1, and 2 M guanidine are shown in Supplemental Figure S1A.”. As seen in this supplemental figure, the WMF red shift is much more pronounced than the increase in fluorescent intensity at higher wavelengths. Instead, we added another analysis method using the ratio of just two wavelengths, described in the text: “An alternate measure of unfolding using the Trp emission ratio at 355/350 nm yielded similar results as the WMF plot; although, the unfolding EC50 values were shifted to lower guanidine, and the ΔC was harder to unfold than the WT isoform using this measure (Supplemental Figure S1B, EC50 = 0.86 and 1.09 M guanidine for the WT and ΔC isoforms, respectively)..”

- In the results corresponding to figure 2, authors mutated Trp8 to Phe, Leu and Ala to discuss the role of the aromatic residue as in the N-terminus unfolding. Trp is an aromatic/amphipathic residue, and it is changed to Phe (aromatic/ hydrophobic) and Leu/Ala (hydrophobic). More disruptive Trp8 changes are needed to clarify the key role of Trp8. Mutations as Trp8 to some polar residue (as Asp and/or Lys and/or Ser) and less bulky residue (Gly) look essential to establish the proper function of the Trp8 into the native structure. Finally, more discussion is needed for mutation Trp8 to Tyr or at least, hypothesize what happens in their model when substitution is performed.

**Response:** We prepared 3 substitution mutations for Trp8, showing that even a small hydrophobic replacement W8A leads to easier unfolding in guanidine and functional rescue ΔC truncation. We don’t think much more will be learned by making additional substitution mutations. Thus, we added the following to the Discussion to address this issue: “We speculate that less conservative substitutions of W8 with charged or polar amino acids or with the even smaller hydrophobic Gly residue would also disrupt the stability of apoA1’s helical bundle and rescue the dysfunction of the ΔC isoform similar to the W8A or ΔN isoforms.”

- It is a clear effect that deletion of the first eight residues into ΔC isoform affects into the unfolding process of the helical bundle. Manuscript would improve if the Δ1-7 and Δ1-8 could be performed into the WT protein and observe the possible effect and comparison with the isoform Δ1-8 ΔC.

**Response:** An even larger deletion in the ΔN isoform has partial to full activity compared to the WT isoform. Thus, we did not expect the Δ1-7 and Δ1-8 isoforms by themselves to have any disrupted activity, and thus we did not prepare these deletions outside of the context of the dysfunctional ΔC isoform.

Minor revisions:

- Authors proposed in the abstract part “we demonstrate that apoA1 lipidation can occur when the barrier to this bundle unfolding is lowered”. Authors should clarify the term “barrier”, if is from a “thermodynamic or energetic” barrier or another type.

**Response:** In the abstract, we specify that this is a “thermodynamic barrier”, and we added to the introduction a sentence describing our prior kinetic and thermodynamic study of the unfolding of the WT, ΔN, ΔC, and ΔN/C isoforms: “We previously demonstrated that the free energy required for guanidine unfolding of apoA1 isoforms is ranked ΔC > WT > ΔN/C > ΔN (16).”

- Introduction lane 2-3: in vivo should be in italics.

**Response:** corrected.

- In the introduction, it is stated “the C-terminal truncation of residues 183-243 (called hereafter the ΔC - isoform), but authors used a C-terminal truncation of residues of 185-243. Authors should explain why residues 183-184 were not deleted and its possible influence.

**Response:** we apologize for the confusion that we created about the endpoint of the ΔC isoform! We re-checked the amino acid sequence of this isoform, and the last amino acid residue it contains is residue 184, thus, we corrected the manuscript to reflect that the ΔC isoform truncates residues 185-243.

- Assays with ΔC and ΔN are well-designed and provided information but more discussion is needed. Specifically, authors should discuss why ΔC is not “more resistant” to unfolding as WT isoform.

**Response:** Although the WMF data plotted in revised Figure 1D did not distinguish between the WT and ΔC isoform, the new supplemental figure S1B shows that the ΔC isoform was more resistant to guanidine unfolding as does the thermal denaturation shown in Figure 1E, F. With this data presented in the revised Results section, along with the correction that reads: “Thermal denaturation showed that the WT isoform was slightly easier to unfold than the ΔC isoform, thus showing that the deletion of the C-terminus stabilized the folded state (Figure 1F)”, we no longer see the need to discuss why the ΔC is not more resistant to unfolding as the WT isoform.

- In Figure 1, it is confusing what black box is. It should be explained, at least, in Figure Legend 1.

**Response:** The legend to Figure 1 was revised to define the black box: “**B.** Cholesterol efflux from ABCA1-induced RAW264.7 cells to different apoA1 isoforms, as indicated, or in the absence of any acceptor (clear bar black outline).”

- Authors mutated Leu 38 and Met 112 to create the 38C/112C helix bundle. Authors should explain better why these residues are chosen as in a quick look of the structure there are more possible candidates.

**Response:** In the revised Results section, we provide a better justification for choosing these residues: “To test the role of unfolding of the helical bundle on apoA1’s lipidation, we used site directed mutagenesis to replace residues Leu38 and Met112, which in the crystal structure (6) are predicted to be only 3.4 angstroms apart and at the other end of the helical bundle from Trp8, with Cys residues to create the 38C/112C helix bundle locked apoA1 isoform (Figure 3A).”

- In figure 3E, black box should be explained at least in Figure Legend 3.

**Response:** The legend was revised: “**E.** Cholesterol efflux from ABCA1-induced RAW264.7 cells to different apoA1 isoforms, as indicated, or in the absence of acceptor (black bar) (average + SD; ***, p<0.001 by one way ANOVA with Dunnett’s posttest compared to WT apoA1).

- In figure 4B, black line is not explained or mentioned.

**Response:** The revised 4B legend reads: “**B.** DMPC MLV clearance by apoA1isoforms, or in the absence of apoA1 (black); same colors as in panel A.” The legend to 4C was also clarified: “**C.** Cholesterol efflux from ABCA1-induced RAW264.7 cells to different apoA1 isoforms or in the absence of any acceptor (clear bar black outline), same colors as in panel A (average + SD; ***, p<0.001 by one way ANOVA with Dunnett’s posttest compared to WT apoA1).”

- Page 12, authors mentioned “Thus, like Trp8, D18 and ΔN/C isoforms….”. It is not clear what D18 is as there are not results refereed to it. Authors should clarify the role of D18 in this context.

**Response:** We apologize for our typos! The corrected sentence reads: “Thus, like the W8A, Δ1-8, and ΔN/C isoforms, these specific proline substitutions promote the helical bundle unfolding and rescue the activity deficits of the ΔC isoform, indicating that the C-terminus is dispensable for apoA1 lipidation when the helical bundle is destabilized.”

- Page 13, authors enlisted a large number of cross links that are not relevant in the discussion part or at least in the main text. It would be more illustrative as supplementary or in a table.

**Response:** We removed the crosslinked exact locations from the text and just refer to the 24 distinct crosslinks between the N- and C-termini, among four independent studies.
