# Supplementary material for: First eight residues of apolipoprotein A-I mediate the C-terminus control of helical bundle unfolding and its lipidation
Source: PLoS One. 2020 Jan 16;15(1):e0221915. doi: 10.1371/journal.pone.0221915 (PMC6964839; doi:10.1371/journal.pone.0221915)
Supplement: Attachment — Submitted filename: Response to review 121019.docx [file pone.0221915.s004.docx]

PONE-D-19-23187R1

First eight residues of apolipoprotein A-I mediate the C-terminus control of helical bundle unfolding and its lipidation

Response to Reviewers:

We thank the editor and the reviewers for their comments, and we have performed additional studies and analysis to address their concerns.

**Editor:**

Both reviewers agree in the manuscript improvement, but to defend the final mechanistic model displayed in Figure 6 the oligomerization control is essential. This control was requested in the first review round and could easily provide it. The study deserves publication in PLoS One but only once this control is provided. Therefore, we invite you to submit a revised version of the manuscript that addresses specifically this point.

**Response:** This is an excellent point, reflecting the last substantive comment of reviewer 1. We copy here our response to Reviewer 1 for this point.

We showed in Fig 1A that all of our recombinant apoA1 isoforms are dimers in solution. Thus the effects of different apoA1 isoforms on DMPC clearance and cholesterol efflux represent effects on dimers. The guanidine and heat denaturation studies may reflect, in part, dimer dissociation or N-and C-terminal inter-molecular interaction. As the reviewer states, we cannot distinguish intramolecular vs. intermolecular effects. Our model shown in Fig. 6 is based upon the previously published consensus model, which was only constructed for an apoA1 monomer. We realize that the N- and C-terminal interaction shown in Fig 6 could be due to intermolecular interactions, rather than the intramolecular interaction as shown. Thus, we have modified the discussion section as follows: “Our model in Fig 6 is based on the monomeric apoA1 consensus model [7]; however, all of the interactions between the N- and C-termini may in fact be due to intermolecular interactions in dimers. The crystal structure of the ΔC isoform shows two antiparallel dimers, with a hinge region that was modeled to fold back upon itself to form a monomer with some intramolecular surfaces replaced by intermolecular surfaces [6]. All of the studied isoforms are dimers, but our denaturation studies cannot distinguish between inter- and intra-molecular interactions involved in unfolding the hairpin bundle.”

**Reviewer #1:**

**Comment:** This reviewer is concerned about the conformational state and energy of the deletion mutants that the authors used to build their hypothesis. I understand that the goal of the study is not to describe the effect of the deletion mutants on the distribution of populations of ApoA1 conformations. But since the authors finally build a mechanistic model on monomeric ApoA1 (Figure 6), they must show that the effects on stability and unfolding presented in the study account on the same protein species as the WT. In this reviewer’s opinion, they still fail to do so. All the protein constructs are shown to be dimers. Different values of fluorescence maxima at 0 GdmHCl may reflect that deletion mutants are partially unfolded or they adopt a molten globule state. This reviewer asked the authors to follow unfolding at a single wavelength as it is the only method to quantitatively monitor protein denaturation and determine the fraction of folded and unfolded protein at each GdmHCl concentration. This reviewer is unable to find the solicited analysis in the author’s reply. The dependence on the concentration argued by the authors is true, but the authors claim they are using the same protein concentrations in all the fluorescence experiments.

**Response:** We analyzed the WMF data using a single wavelength, and we have added this data as Supplemental Figure S1B. We added to the text” “An alternate measure of unfolding using the Trp emission at 345 nm yielded similar results as the WMF plot; although, the unfolding EC50 values was shifted to a lower guanidine concentration for the ΔC isoform (Supplemental Fig S1B, EC50 = 1.12, 0.76, 1.06, and 0.31 M guanidine for unfolding of the WT, ΔN, ΔC, and ΔN/C apoA1 isoforms, respectively). The % folded by this measure is shown in Supplemental Fig S1C.” We did not favor this analysis because we are performing the studies at the same μg/ml protein concentration, and since the isoforms have different lengths and different number of Trp residues (4 or 3), the absolute values are somewhat meaningless, although the changes with guanidine reflect what was observed using the WMF measure, which is the standard in the apoA1 unfolding field. We normalized this as % folded in Supplemental Fig S1C.

**Comment:** Regarding CD measurements, it is true that quantification of secondary structure based on changes in ellipticity may not be very robust. Authors claim that CD follows secondary structure and the study aims to describe changes in tertiary structure. This reviewer is concerned about the effect that quaternary structure may induce in the fluorescence data since it has been completely neglected from the interpretation. CD would be ideal to show if the deletion mutants adopt a molten globule conformation at 0 M GdmHCl. Thermal denaturing (particularly DSC) would inform on the possible sequential dimer dissociation-unfolding. The authors added a new line showing the values of secondary structure elements. They must show the CD spectra at increasing concentrations of GdmHCl.

**Response:** We performed new CD assessment at various guanidine concentrations and added a Supplemental Figure and the following text: “We performed circular dichroism on these four isoforms and determined α-helicity of 46.0%, 50.7%, 51.9%, and 50.7% for the WT, ΔN, ΔC, and ΔN/C isoforms, respectively, which are similar to values previously reported for similar recombinant apoA1 isoforms [19,20]. Upon increasing guanidine, we observed % α-helicity decreasing with the ΔN and ΔN/C more sensitive to guanidine, similar to the WMF assay (Supplemental Figure S1D, EC50 = 1.06, 0.48, 0.97, 0.20 M guanidine for the WT, ΔN, ΔC, and ΔN/C isoforms, respectively).”

**Comment:** All in all, it is still not clear whether the destabilizing effects on the helix bundle upon lipidation of the C-terminal tail happens intramolecularly or intermolecularly. The data presented by the authors is insufficient to answer this question. The authors still should reply or propose an explanation why mutants only partially recover the ability to solubilize DMPC (New Figure 1B).

**Response:** We showed in Fig 1A that all of our recombinant apoA1 isoforms are dimers in solution. Thus the effects of different apoA1 isoforms on DMPC clearance and cholesterol efflux represent effects on dimers. The guanidine and heat denaturation studies may reflect, in part, dimer dissociation or N-and C-terminal inter-molecular interaction. As the reviewer states, we cannot distinguish intramolecular vs. intermolecular effects. Our model shown in Fig. 6 is based upon the previously published consensus model, which was only constructed for an apoA1 monomer. We realize that the N- and C-terminal interaction shown in Fig 6 could be due to intermolecular interactions, rather than the intramolecular interaction as shown. Thus, we have modified the discussion section as follows: “Our model in Fig 6 is based on the monomeric apoA1 consensus model [7]; however, all of the interactions between the N- and C-termini may in fact be due to intermolecular interactions in dimers. The crystal structure of the ΔC isoform shows two antiparallel dimers, with a hinge region that was modeled to fold back upon itself to form a monomer with some intramolecular surfaces replaced by intermolecular surfaces [6]. All of the studied isoforms are dimers, but our denaturation studies cannot distinguish between inter- and intra-molecular interactions involved in unfolding the hairpin bundle.”

**Response:** This reviewer is still concerned about the difference between the WT, ΔN, and ΔN/C isoforms in the DMPC clearance assay in Fig 1B. The DMPC clearance assay is subject to variance due to the rapid initial clearance rate, the position of samples in the 96-well plate, and difference in time to read the different apoA1 isoforms. These affect the 0 time read, which is used to normalize all of the subsequent time points. We use this assay more as a qualitative vs. quantitative assay to determine if apoA1 is active. The cholesterol efflux assay is more physiological and shows that the ΔN and ΔN/C isoforms behave similar to the WT isoform.

**Comment:** This reviewer suggests further editing of the text; some minor mistakes are still present.

**Response:** we reviewed the manuscript again to identify and fix any remaining errors.
